# Developing a Common Global Baseline for Nucleic Acid Synthesis Screening

**DOI:** 10.1089/apb.2023.0034

**Published:** 2024-06-20

**Authors:** Nicole E. Wheeler, Sarah R. Carter, Tessa Alexanian, Christopher Isaac, Jaime Yassif, Piers Millet

**Affiliations:** ^1^ Institute of Microbiology and Infection, University of Birmingham, Birmingham, United Kingdom.; ^2^ Science Policy Consulting LLC, Arlington, Virginia, USA.; ^3^ International Biosecurity and Biosafety Initiative for Science (IBBIS), Geneva, Switzerland.; ^4^ Nuclear Threat Initiative, Washington, DC, USA.

**Keywords:** biosecurity, nucleic acid synthesis, synthesis screening, synthetic DNA, bioinformatics

## Abstract

**Introduction:**

Nucleic acid synthesis is a powerful tool that has revolutionized the life sciences. However, the misuse of synthetic nucleic acids could pose a serious threat to public health and safety. There is a need for international standards for nucleic acid synthesis screening to help prevent the misuse of this technology.

**Methods:**

We outline current barriers to the adoption of screening, which include the cost of developing screening tools and resources, adapting to existing commercial practices, internationalizing screening, and adapting screening to benchtop nucleic acid synthesis devices. To address these challenges, we then introduce the Common Mechanism for DNA Synthesis Screening, which was developed in consultation with a technical consortium of experts in DNA synthesis, synthetic biology, biosecurity, and policy, with the aim of addressing current barriers. The Common Mechanism software uses a variety of methods to identify sequences of concern, identify taxonomic best matches to regulated pathogens, and identify benign genes that can be cleared for synthesis. Finally, we describe outstanding challenges in the development of screening practices.

**Results:**

The Common Mechanism is a step toward ensuring the safe and responsible use of synthetic nucleic acids. It provides a baseline capability that overcomes challenges to nucleic acid synthesis screening and provides a solution for broader international adoption of screening practices.

**Conclusion:**

The Common Mechanism is a valuable tool for preventing the misuse of synthetic nucleic acids. It is a critical step toward ensuring the safe and responsible use of this powerful technology.

## Introduction

Over the past 20 years, increased access to synthetic nucleic acids, primarily from commercial providers, has fueled global progress in biotechnology, helping to address challenges in health, climate change, food security, and economic development. However, low-cost, globally distributed synthesis capabilities make it easier for bad actors to access nucleic acids that could produce dangerous biological agents, with potentially catastrophic consequences for public health and safety.

No country legally requires that nucleic acid synthesis orders be screened to ensure that pathogen and toxin sequences are not inadvertently sold to malicious actors. Voluntary practices for screening customers and sequences ordered are outlined in the Harmonized Screening Protocol^1,2^ of the International Gene Synthesis Consortium (IGSC), an industry group that represents a majority of the global synthesis market, as well as the U.S. Department of Health and Human Services (HHS) Screening Framework Guidance, issued in 2010 and updated in 2023.^3^ Recent advances in artificial intelligence (AI) have renewed attention on nucleic acid synthesis screening,^4,5^ due to concerns about AI-designed bioweapons, and new frameworks and incentives for screening are being implemented in the United States.^6^

Nucleic acid synthesis screening offers additional benefits beyond preventing the malicious misuse of DNA. Screening also has the potential to prevent the conduct of pathogen research in laboratories with inadequate biosafety measures, and to improve public trust in the bioeconomy by demonstrating a commitment to responsible practices.

There have been some efforts to promote and promulgate nucleic acid synthesis screening internationally. Meetings of the Biological Weapons Convention (BWC) have referenced the IGSC and their Harmonized Screening in background documents, including in a 2009 background document, 2012 BWC Meeting of Experts presentation, and 2018 Working Paper submitted by the U.S. government.^7–9^ From 2012 to 2013, a series of meetings of states, international organizations, and industry, held in Germany and China, discussed nucleic acid synthesis screening.^10^ The issue was also picked up in the 2022 Global Guidance Framework for the responsible use of the life sciences produced by the World Health Organization.^11^ The International Standards Organization is currently working on Requirements for the Production and Quality Control of Synthesized Gene Fragments, Genes, and Genomes.^12^

Despite these efforts, there remains a lack of internationally recognized standards and frameworks for synthesis screening. There is a need to address challenges related to the cost of technical development and maintenance of tools and resources, maintaining confidentiality between providers and customers, and ensuring the trustworthiness of the synthesis screening system. Here, we describe the Common Mechanism for DNA Synthesis Screening (Common Mechanism) as a baseline screening system—one that aims to provide a minimal capability, which overcomes these challenges, and provides a solution for broader international adoption of screening practices.

## Barriers to the Adoption of Nucleic Acid Synthesis Screening

Nucleic acid synthesis screening practices are challenging to adopt and maintain.^13–15^ These challenges put significant pressure on nucleic acid providers who already screen, and make it difficult to expand nucleic acid synthesis screening practices to those who do not.

### Reducing Costs and Meeting Commercial Needs for Synthesis Providers

Developing tools and resources for nucleic acid synthesis screening is a complex and challenging task. To flag potential sequences of concern in ordered nucleic acids, providers must either acquire in-house expertise in a wide range of fields, including molecular biology, bioinformatics, and computer science, or take on the ongoing operating cost of purchasing commercially available software. The price per base of synthesis is decreasing and the volume of orders is increasing, making screening an increasingly difficult economic burden for nucleic acid providers.^13,15,16^

At the same time, commercial providers see tremendous value in keeping their screening mechanisms in-house. A customer's nucleic acid sequences can be highly sensitive intellectual property. For this reason, nucleic acid providers generally provide customers with explicit contractual agreements that they will not share information about the customer or their ordered nucleic acid sequences with third parties. These agreements are seen by some nucleic acid providers as critical to their business, making it difficult for them to fully off-load sequence screening to third parties.

### Building International Trust in Screening Practices

To date, there has been limited international collaboration on nucleic acid synthesis screening policies and practices. IGSC members include synthesis companies headquartered in Austria, China, France, Japan, South Korea, the United Kingdom, and the United States, but the IGSC's Harmonized Screening Protocol is a set of practices, rather than a practical tool, and the organization has no full-time employees nor funding to maintain the Registered Pathogens Database.

The U.S. government has invested significant resources in developing databases of sequences of concern and nucleic acid sequence screening tools, but this funding had strong ties with U.S. national defense and intelligence sectors (e.g., Intelligence Advanced Research Projects Activity's FunGCAT program), and it has yielded resources that were not widely available and were offered by organizations with strong ties to the U.S. government. In addition to export controls that restrict international sharing of these resources, the closed-door development process has made it difficult for a wide range of international stakeholders to benefit from and fully trust these advances.

### Integrating Screening into Benchtop Devices

In the near future, it is likely that a wider range of benchtop nucleic acid synthesis devices will become available, which could drive some nucleic acid synthesis away from centralized providers.^17^ The updated HHS Screening Framework Guidance recommends that benchtop device manufacturers integrate sequence screening into these devices, and manufacturers are grappling with how to do so in an effective and efficient way.

There are many different ways that sequence screening could be incorporated into the benchtop synthesis workflow, and most manufacturers anticipate screening by a “phone home” approach in which the device sends sequences to the manufacturer or a cloud-based screening service before printing the nucleic acid sequence.^17^ This type of screening will raise similar challenges to those seen by traditional nucleic acid providers. If screening is integrated into the device itself there will be an increased need for unambiguous and automated systems for flagging sequences.

## Building a Baseline Screening System

The Common Mechanism is designed to address the challenges described above, and act as a global baseline for nucleic acid synthesis screening that is cost-effective, meets commercial users' needs, is easily adopted around the world, and is adaptable to benchtop DNA synthesis devices. The Common Mechanism was proposed in 2020 by an international working group organized by the Nuclear Threat Initiative (NTI) and the World Economic Forum,^18^ which also called for an international entity that will house the mechanism, promote its adoption, and work to establish global norms for nucleic acid synthesis screening.

Later that year, NTI launched a Technical Consortium to build the Common Mechanism, consisting of African, Asian, European, and North American experts from industry, academia, philanthropy, and international organizations.^19^ From 2020 to 2023, this Consortium guided the design of the baseline screening process described below. In February 2024, a new, international organization, the International Biosecurity and Biosafety Initiative for Science (IBBIS) (https://ibbis.bio/), was launched to act as a long-term home for the Technical Consortium and Common Mechanism, as well as to support a broader range of related initiatives for advancing biosecurity and biotechnology governance.

The Common Mechanism's sequence screening databases and algorithms are currently in beta testing, with a public release planned for 2024 to coincide with the formal launch of IBBIS. The Common Mechanism will ultimately include a customer screening framework, using best practices and resources identified by the Technical Consortium, but this article will focus on the nucleic acid screening component of our work.

The Common Mechanism is based on best practices for sequence screening. The HHS Screening Framework Guidance and the IGSC recommend screening of sequences that are ≥200 nucleotides in length to determine if the sequence is “best match” to a sequence from a regulated pathogen or toxin. The recently updated Screening Framework Guidance calls on nucleic acid providers to begin screening sequences as short as 50 nucleotides within 3 years, and further recommends nucleic acid providers screen for a broader range of “sequences of concern,” defined as “sequences known to contribute to pathogenicity or toxicity, even when not derived from or encoding regulated biological agents.”^3^

Most nucleic acid providers must also comply with regulations that require them to obtain an export license for some sequences. For example, in countries that comply with the Australia Group export regime, nucleic acid sequences from a listed pathogen that may “endow or enhance” pathogenicity are subject to export control.

**Figure 1.**
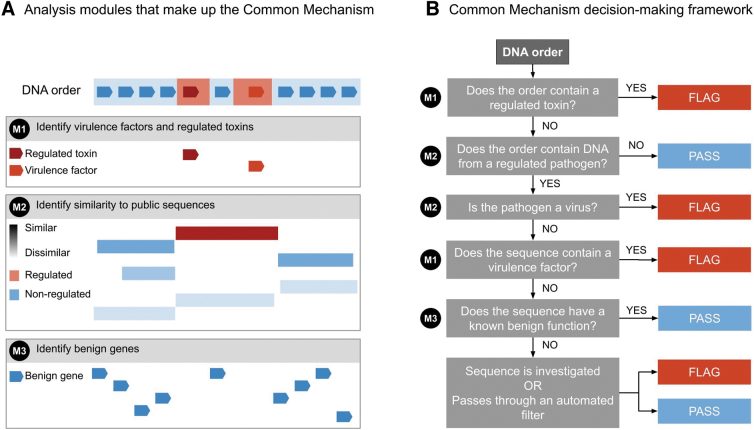
**(A)** Analysis modules that make up the Common Mechanism. **(B)** Sequence screening decision-making framework employed by the Common Mechanism to determine whether a sequence is flagged, or if it can pass. For each stage, if a sequence is not flagged or cleared, then it will move on to the next stage for more querying.

Incorporating these best practices for nucleic acid sequence screening, the Common Mechanism will screen sequences that are ≥50 nucleotides, and flag both “sequences of concern” and sequences that may be subject to export controls (Figure 1).

### Identifying Sequences of Concern

The first module of the Common Mechanism (M1 in Figure 1A) compares order sequences against a “biorisk” database equipped with probabilistic models capturing profiles of sequences of concern. These models are trained to recognize a wide range of both naturally occurring and engineered variants of these sequences, ensuring they can detect diverse threats, even those designed to avoid standard screening. The initial “biorisk” database used by the Common Mechanism draws only from existing, publicly available databases of sequences known to be associated with toxicity or pathogenicity.

This list is further limited to only those sequences that are found in regulated, listed pathogens and toxins. This limited biorisk database allows the Common Mechanism to flag sequences that are well established, transparently sourced, and represent some level of international consensus. Matches to regulated toxins in the biorisk database are always flagged.

### Identifying Taxonomic Best Matches

The second screening module employs a “best match” method (M2 in Figure 1A). This involves comparing the ordered nucleic acid sequence against publicly available DNA and protein sequences to retrieve the organism with the most closely matching DNA or protein sequence. The identified matches are then cross-referenced with international control lists of organisms to see if it is included on these lists. This process is consistent with recommendations from the HHS Screening Framework Guidance and current industry best practices. If the taxonomic best match is to a regulated viral pathogen, or the sequence was flagged as a sequence of concern by the first module, the order will be flagged.

### Identifying Benign Genes

The third screening module evaluates nucleic acid sequences that are found to be “best match” to a regulated, nonviral organism, and identifies those with a known benign function (M3 in Figure 1A), such as sequences supporting essential cellular processes, as these can be exported without an export license and do not pose a risk of misuse. Benign sequences have been identified by sourcing genes from (1) databases of nucleic acid sequences with shared ancestry and function^20^ that are found in thousands of bacterial species; (2) RNA sequences that participate in processes essential for life^21^; and (3) sequences submitted to the iGEM synthetic biology parts registry that had no safety flags attached to them.^22^ Sequences found to be benign can pass through screening without being flagged, consistent with the HHS Screening Framework Guidance and current best practices.^3,16^

### Processing Results and Flagging Orders for Human Review

The Common Mechanism will flag all sequences from regulated toxins and viruses, as well as “virulence factors” (genes involved in causing disease) from nonviral regulated pathogens (Figure 1B); this approach is consistent with the current export controls. Many sequences found in regulated pathogens are neither known to be benign nor known to be virulence factors (indicated in the final box in Figure 1B); nucleic acid providers can choose whether to further investigate these orders based on the provider's resources and risk tolerance. A responsible nucleic acid provider might want to check with export control officials for orders containing sequences of unknown function in nonviral pathogens, but there are no established best practices. These types of sequences are likely to be a very small fraction of orders.

### Advantages of the Common Mechanism

The Common Mechanism was designed to address many of the challenges to synthesis screening described above. To reduce costs and meet the needs of commercial vendors, it will be provided as a free, open-source software tool, and the initial versions of the databases used to identify toxins, virulence factors, benign genes, and regulated pathogens will be released publicly. This will allow all nucleic acid synthesis companies to adopt baseline screening practices at no cost beyond computing and to maintain screening in-house, without the need to send their customers' sequences outside of the company.

In addition, a key goal of the Common Mechanism is to reduce ambiguity and the number of false positives by only flagging sequences associated with pathogens and toxins listed under established regulations. This clarity will reduce the need for costly follow-up review of flagged sequences. Lowering these cost barriers will enable companies that currently do not screen to adopt a baseline level of screening.

For companies that already have screening systems in place, the Common Mechanism has been designed to allow integration of external databases (beyond the baseline databases, as described above) and substitutions of each of the analysis modules (Figure 1A), so companies can integrate in-house resources they have already invested in. This approach allows each nucleic acid provider to work from a common baseline, and develop customizations or improvements that better meet their needs. For example, some responsible nucleic acid providers currently screen a broader range of sequences of concern than the Common Mechanism and invest in screening improvements, and this modular approach supports those providers. Sharing these advances with the community helps enable them to contribute to development of best practices for the industry.

To ensure that the Common Mechanism could be used and trusted throughout the world, we led a development process that included international engagement through the Technical Consortium, which included African, Asian, European, and North American experts. As the Common Mechanism took shape, progress updates were openly shared through a number of international events across Asia, Europe, and North America—including Carnegie India's Global Technology Summit, the Ninth Review Conference of the Biological Weapons Convention, and the Global Biofoundries Alliance annual meeting (Table 1).

**Table 1.** International events briefed on the Common Mechanism

***Meeting***	***Location***	***Date***
Biosecurity Working Group of the G7 Global Partnership against the Spread of Weapons and Materials of Mass Destruction	Berlin, Germany	October 2022
7th Annual Global Technology Summit	New Delhi, India	December 2022
Biological Weapons Convention Ninth Review Conference	Geneva, Switzerland	December 2022
Munich Security Conference	Munich, Germany	February 2023
SynBioBeta Global Synthetic Biology Conference	Oakland, USA	June 2023
Biosecurity Innovation and Risk Reduction Initiative annual meeting	Cambridge, England	June 2023
BioRisk Association of the Philippines annual meeting	Manila, Philippines	July 2023
Global Biofoundaries Alliance annual meeting	Copenhagen, Denmark	September 2023
Paris Peace Forum	Paris, France	November 2023

This transparency has allowed the Common Mechanism to benefit from diverse perspectives on technical priorities and to reassure international stakeholders, including governments, that the Common Mechanism represents some level of international consensus for nucleic acid sequence screening. Making both the contents of the databases and the source code for the algorithm available ensures that stakeholders can trust that the Common Mechanism will behave as expected, will not share data with any third parties, and will not flag sequences for unexpected reasons.

The Common Mechanism can be adapted to be fully automated with its dual emphasis on reducing ambiguities and flagging a baseline level of sequences of concern—making it a useful tool for use with benchtop devices, especially when screening is distributed rather than cloud based. For example, a device could be configured, so that it does not synthesize sequences that are flagged during screening; legitimate users of those sequences would need to order them from another commercial provider or provide an authentication key that allows them to synthesize specific sequences of concern. To ensure applicability of the Common Mechanism for this context, benchtop device manufacturers have been a key part of the Technical Consortium,^19,23^ and ongoing engagement with these companies will be critical for continuing to develop best practices in this area.

Many benchtop devices are excluded from screening at present, as they produce fragments <200 nucleotides, but this will change as devices improve and screening expands to sequences as short as 50 nucleotides. The proactive engagement of this industry in developing these screening tools and practices will ensure that they are effective and fit-for-purpose.

In some cases, addressing these challenges requires trade-offs. In particular, the Common Mechanism was designed to reflect international consensus and to build trust through collaborative, transparent development. These design criteria limit screening to sequences with well-established links to pathogenicity found in regulated pathogens, which only captures a fraction of sequences that could be misused to cause harm. As discussed below, future versions of the Common Mechanism may move beyond this baseline approach, but doing so will either require significant international consensus building or will be offered as additional features on top of the baseline screening mechanism.

Openly sharing the Common Mechanism raises the possibility that malicious actors could try to design sequences to evade it. The screening mechanism captures a range of biologically viable variants of established biorisk sequences, making evasion difficult (a technical paper is forthcoming). This open-source approach also enables more robust third-party review and testing to improve the mechanism over time. Analogous open-source approaches have been used effectively within the cybersecurity community and are likely to be highly effective in this context as well. Fundamentally, because it will enable broader adoption of screening practices, the Common Mechanism can improve overall biosecurity notwithstanding the tradeoffs involved in taking an open-source approach.

## Outstanding Challenges

### Expanding Beyond Regulated Pathogens and Toxins

There has been substantial interest in developing databases and nucleic acid sequence screening systems that flag a wider range of sequences of concern.^15,24,25^ Indeed, the updated HHS Screening Framework Guidance recommends that best practices be developed to expand sequence screening to capture sequences “known to contribute to pathogenicity or toxicity” not derived from regulated pathogens and toxins.

This goal is commendable, and efforts to develop and share these practices will undoubtedly improve nucleic acid sequence screening and advance biosecurity. However, as a first step toward more universal screening, the Common Mechanism can play a critical role in establishing a shared baseline. In the future, as international consensus is reached on broader definitions for sequences of concern, the Common Mechanism can be updated to incorporate these consensus sequences and raise the baseline of screening practices.

### Updating Screening as Science and Policy Develop

Successful nucleic acid sequence screening requires databases that are frequently updated. Our shared scientific understanding of what should be considered a sequence of concern or a benign sequence is constantly evolving, yet research results are not routinely integrated into public databases of virulence factors, creating a risk that screening will miss known hazards. Alongside this challenge, screening must account for policy processes that update lists of regulated pathogens and toxins. IBBIS can act as a natural home for international efforts to develop and maintain updated databases that reflect both scientific understanding and regulation in practice. Continued work by the Technical Consortium, which will be part of IBBIs' activities going forward, will provide the technical expertise needed to drive such a process.

The use of AI for protein design has also raised a critical challenge for nucleic acid synthesis screening because it may enable the design of functional proteins with very low sequence homology to known proteins.^5^

The Common Mechanism's first module is trained to recognize both sequences of concern in the biorisk database and predicted functional variants of them. As additional tools are developed to more accurately predict whether a sequence has a function of concern, the Common Mechanism databases can be updated to incorporate these advances. Development of sequence-to-function prediction tools will require engagement from protein designers, nucleic acid providers, sequence screening and biosecurity experts, and others. To maintain trust and support international adoption, it will be critical that this process includes international stakeholders, and IBBIS will work to facilitate this process.

### Limiting Information Hazards from Shared Sequence Databases

The intent of nucleic acid synthesis screening is to ensure that a malicious actor is not provided the nucleic acids necessary to build dangerous pathogens or toxins. However, there is no consensus, scientifically or legally, on which nucleic acid sequences might constitute a “sequence of concern” that could be misused.^25,26^ This uncertainty and ambiguity about sequences of concern is a key challenge for nucleic acid providers, driving up costs for bioinformatic review and requiring each company to make its own judgment about how to interpret these recommendations and rules,^16^ (“Progress And Prospects For a Nucleic Acid Screening Test Set,” submitted to the same issue). Creating a shared database of “sequences of concern” helps alleviate this problem.

Highly curated databases of sequences of concern, if shared widely, could create an “information hazard” by informing malicious actors of potential avenues to cause harm.^27^ However, as described above, the initial biorisk database developed for the Common Mechanism is a baseline resource that is limited in scope. Because it only uses protein sequences that are already listed in publicly available biorisk databases and that are found in regulated pathogens and toxins, the release of the biorisk database poses only a minimal information hazard.^23^

Future databases that incorporate more novel sequences and functional variants may be more appropriately released only to approved users such as nucleic acid providers and screening tool developers. This approach is consistent with the HHS Screening Framework Guidance, which calls for precautions when sharing databases of sequences of concern that include unregulated pathogens and toxins.

## Conclusions

The Common Mechanism will help expand global nucleic acid synthesis screening through its transparent and collaborative development process and its broadly applicable baseline screening approach. However, additional incentives are needed, particularly outside of the United States, to ensure that nucleic acid providers adopt and continue to improve screening practices.

In the United States, the recently released “Executive Order on the Safe, Secure, and Trustworthy Development and Use of Artificial Intelligence”^6^ states that agencies that fund life sciences research should require that funded research only purchase nucleic acid sequences from providers that conduct customer and sequence screening. This is a welcome development and will provide a strong incentive for screening. The AI Executive Order further directs agencies to develop resources to support screening, including a database of sequences of concern and standards and metrics for screening practices.

Currently, companies develop these lists independently, which raises the risk that a customer could submit an order to different companies in the hopes that one will not flag their sequence.^13^ Established standards would help address this vulnerability. Ongoing efforts to support benchmarking performance of screening tools, including the Common Mechanism, are underway (see also Progress and Prospects for a Nucleic Acid Screening Test Set, submitted to the same issue), and could contribute to development of standards and auditing regimes.

As U.S. government agencies work to comply with the AI Executive Order, it will be important for them to collaborate with international partners, including IBBIS, to learn from previous efforts and to ensure that a baseline level of screening practices can be established globally. Governments outside of the United States, including the United Kingdom, are considering guidance or regulations for screening by nucleic acid providers.^28^ Harmonizing requirements across countries will reduce ambiguity, support compliance, and prevent market disincentives for screening practices.^15^ We anticipate that the Common Mechanism can help fill this need and hope that national governments will find it useful.

There are several additional ways that screening practices can be supported internationally. Binding international agreements focused on weapons of mass destruction, including the BWC and UN Security Council Resolution 1540, could endorse, or perhaps even require, including nucleic acid synthesis screening in national implementation measures. Guidance could also be issued through the World Health Organization or regional organizations such as the Africa Centers for Disease Control and Prevention or the Pan American Health Organization. Development of standards through the International Standards Organization could include screening practices, for example, as part of standards currently under development for nucleic acid synthesis.

A variety of approaches to expanding nucleic acid synthesis screening internationally exist, and many of these are mutually reinforcing. IBBIS will be well positioned to develop a “Seal of Approval” for nucleic acid providers that comply with a baseline standard of screening practices, which could provide a reputational boost to responsible companies and form the basis for a future international certification system for nucleic acid providers. By establishing a transparent, baseline level for nucleic acid sequence screening, the Common Mechanism supports the development of these standards, certification systems for nucleic acid providers, and more binding rules, including international commitments and regulations at the national level.

In conclusion, the Common Mechanism is a valuable tool for preventing the misuse of synthetic nucleic acids. It is a critical step toward ensuring the safe and responsible use of this powerful technology.
